# Homozygous TRPV4 Mutation Broadens the Phenotypic Spectrum of Congenital Spinal Muscular Atrophy and Arthrogryposis: A Case Report

**DOI:** 10.7759/cureus.43413

**Published:** 2023-08-13

**Authors:** Elyette Lugo, Eric Graulau, Edwardo Ramos Cortes, Simón Carlo, Norman Ramírez

**Affiliations:** 1 Medicine, Universidad Central del Caribe, Bayamón, PRI; 2 Medicine, Ponce Health Sciences University, Ponce, PRI; 3 Physical Medicine and Rehabilitation, University of Puerto Rico, Medical Sciences Campus, San Juan, PRI; 4 Biochemistry/Pediatrics/Psychiatry, Ponce Health Sciences University, Ponce, PRI; 5 Pediatrics, Mayagüez Medical Center, Mayagüez, PRI; 6 Pediatric Orthopedic Surgery, Mayagüez Medical Center, Mayagüez, PRI

**Keywords:** motor neuron disease, phenotypic variability, arthrogryposis, congenital spinal muscular atrophy, trpv4

## Abstract

Transient receptor potential vanilloid 4 (TRPV4) mutations are known to cause inherited axonal neuropathies and skeletal dysplasia. TRPV4 mutations are associated with distal hereditary motor neuropathies (dHMN), which distinctly involve motor deficits. A 1 ½-year-old boy presented at the clinic with diminished lower limb movement and ambulatory limitations. The patient was born with bilateral knee arthrogryposis and bilateral talipes equinovarus, which required surgical intervention. A gross neurologic exam was unremarkable, with normal vision and hearing. A bone survey radiograph showed no evidence of skeletal dysplasia. Genetic tests revealed a homozygous mutation in the TRPV4 gene (c.281C>T; p.S94L), leading to the diagnosis of congenital spinal muscular atrophy and arthrogryposis (CSMAA). Hence, this presents the first case of CSMAA caused by a TRPV4 mutation (p.S94L), with a different presentation from the one previously described in the literature, thus broadening the phenotypic variability and clinical spectrum of TRPV4 mutations.

## Introduction

The transient receptor potential vanilloid 4 (TRPV4) gene, located on chromosome 12, encodes a cation channel that is selectively impermeable to calcium. It belongs to the transient receptor potential (TRP) family of channels [1, 2]. The TRPV4 protein consists of six transmembrane domains, a cation-conducting pore between transmembrane domains 5 and 6, as well as N-terminal and C-terminal intracellular tails [1]. The TRPV4 channel is recognized as a thermo- and osmo-sensor due to its responsiveness to hypotonic cell swelling and shear stress [1, 2, 3]. Additionally, TRPV4 can be activated by various stimuli, including but not limited to changes in temperature, acidic pH, endogenous ligands, phorbol esters, and arachidonic acid [3, 4, 5]. TRPV4 is involved in numerous physiological functions and is expressed in a wide range of tissues, primarily cartilage and bone [2, 6]. As a result, it plays a significant role in generating Ca^2+^ signals, membrane potential depolarization, and the modulation of chondrogenic bone formation and endochondral ossification [2, 6]. Furthermore, the TRPV4 protein participates in mechanosensation, osmosensation, and nociception/pain in sensory neurons such as the dorsal and trigeminal root ganglia [2, 3, 6].

Mutations in the TRPV4 gene can lead to a wide range of hereditary axonal neuropathies and skeletal dysplasias [1, 2]. These neuromuscular disorders are widely known for causing distal and proximal muscle weakness and wasting, including manifestations such as vocal cord paralysis, absent deep tendon reflexes, sensorineural hearing loss, and essential tremors [4, 5]. Malfunctioning of the TRPV4 channel is also associated with defective bladder voiding, kidney functioning, and regulatory volume decrease in the human tracheal epithelium [4, 6]. These neuromuscular disorders represent a broad clinical spectrum and can be further subdivided into categories such as scapuloperoneal spinal muscular atrophy (SPSMA), autosomal dominant axonal Charcot-Marie-Tooth type 2C (CMT2C), and congenital distal spinal muscular atrophy (dSMA) with overlapping clinical presentations [4, 7, 8].

Bone dysplasias are distinguished by the presence of short stature, kyphoscoliosis, bowing of the extremities, joint contractures, and platyspondyly [6]. Additionally, decreased bone density and irregularities in endochondral ossification, along with areas of stippled calcification in epiphyses and metaphyses, are well-known characteristics [4, 6]. Skeletal dysplasias include metatropic dysplasia (MTD), spondylometaphyseal dysplasia Kozlowski type (SMDK), and autosomal dominant brachyolmia [6, 9, 10, 11].

This report presents a case of a 1 ½-year-old boy with a homozygous TRPV4 mutation (c.281C>T; p.S94L) diagnosed with congenital spinal muscular atrophy and arthrogryposis (CSMAA). This case represents a challenging diagnosis since the patient had no skeletal dysplasia, torticollis, vocal cord paralysis, or any TRPV4-mutation-related symptoms previously mentioned in the literature. Thus, confirming and broadening the phenotypic variability in the presentation of TRPV4 mutations. 

## Case presentation

A male patient born to unrelated, healthy parents of Puerto Rican descent after 39 weeks of gestation was taken to a pediatric orthopedic clinic after six days of life due to ankylosed knees and bilateral talipes equinovarus. He underwent treatment with the Ponseti casting method for two months to correct his bilateral talipes equinovarus. Subsequently, Achilles tendon lengthening surgery was performed on both legs, following established guidelines. Post-surgery, the patient was instructed to wear a Ponseti brace to maintain the correction. However, at the 8-month follow-up, the patient returned to the clinic for evaluation, exhibiting evidence of recurrence of his clubfeet. Due to the recurrence, a period of recasting with Achilles tendon lengthening was deemed necessary.

A year later, the patient demonstrated ambulatory delay due to knee and ankle contractures. At two years of age, the patient could sit independently and walk on his knees. However, he was unable to stand or walk independently. Other than the lower limb clinical manifestations, the patient has normal speech, fine motor and gross motor skills in his upper limbs, normal hearing, and visual development.

Due to his clinical presentation and distinctive facial features, he was evaluated by a neurologist, geneticist, and pediatric physical medical rehabilitation clinic due to suspected geleophysic dysplasia. These facial features included a round face with full cheeks, a small nose with upturned nostrils, a broad nasal bridge, a thin upper lip, and upturned corners of the mouth, in addition to his contractures and limited mobility. However, the bone survey did not show evidence of bone dysplasia. Additionally, the child was assessed by a cardiologist and gastroenterologist to rule out other features of geleophysic dysplasia. The results were within normal limits as the patient did not exhibit any other manifestations associated with this dysplasia, such as heart problems (thickened valves, pulmonary stenosis, atrial septal defect) or an enlarged liver (hepatomegaly).

A skeletal disorder panel was ordered for the subject, which included sequence analysis and deletion/duplication testing of 358 genes. Genomic DNA was isolated from the patient's blood and resulted in a positive finding of a homozygous mutation in the TRPV4 gene (c.281C>T; p.S94L). The parents are heterozygous for the same mutation, without any clinical manifestations. The patient was diagnosed with congenital spinal muscular atrophy and arthrogryposis (CMSAA).

Electromyography studies were performed, revealing evidence of a pure motor neuron disease, ruling out the possibility of a neuropathy (CMT) (see Tables 1, 2, 3). Nerve conduction assessments showed normal sensory nerve parameters in the upper and lower limbs (refer to Table 1). Motor nerve conduction assessment revealed the right (R) motor peroneal motor nerve with normal latency and conduction velocity but decreased amplitude (refer to Table 2). Needle examination of muscles in the lower limbs showed increased insertional activity with denervation potentials, fibrillation potentials with poor recruitment, and mild enlargement of motor units ranging from 2.5 K to 3 K (refer to Table 3).

**Table 1:** Sensory Nerve Conduction Assessment in Median and Sural Nerves ms: millisecond, *µ*V: microvolts, nVxs: nanoVolts second, mA: milliamperes, mm: millimeters, m/s: meter per second.

Test	Stimulation (recording) sites	Latitude (ms)	Amplitude (µV)	Duration (ms)	Area nV×s	Stimulation (mA)	Stimulation (ms)	Distance (mm)	Time, (ms)	Velocity (m/s)	
Right, Median Sensory											
1	wrist	2.6	15.8	1.4	8.8	13	0.1	140	2.6	53.8	
Right, Sural Sensory											
6	1	4.05	13.4	1.7	11.8	13	0.1	140	4.05	34.6	

**Table 2:** Motor Nerve Conduction Assessment: Upper and Lower Limbs ms: millisecond, mV: millivolt, mA: milliamperes, m/s: meter per second.

Test	Stimulation site	Latitude (ms)	Amplitude (mV)	Duration (ms)	Area (mV×ms)	Stimulation (mA)	Stimulation (ms)	Distance (mm)	Time, (ms)	Velocity (m/s)	
Left, Median Motor											
3	wrist	2.9	6.66	4.25	14.9	23	0.2	80			
	elbow	4.8	6.52	5.5	21.4	23	0.2	120	1.9	63.2	
Right, Ulnar Motor											
2	wrist	2.05	7.06	5.55	20.3	15	0.2	80			
	below elbow	3.55	8.98	5.5	26.4	15	0.2	105	1.5	70.0	
Right, Peroneal Motor											
5	ankle	2.7	0.625	4.35	6.6	61	0.2	80			
	fibular head	4.35	0.452	4.85	1.0	80	0.2	95	1.65	57.6	

 

**Table 3:** Electromyography (EMG) Findings in Neurogenic Muscle: A Comparative Analysis of Vastus Lateralis and Tibialis Anterior Muscles +1: persistence of fibrillation potential in at least two areas, +2: persistence of fibrillation potential in three or more areas.

Test	Spontaneous activity	MUP amplitude	MUP duration	MUP polyphasicity	Pattern
Right, Vastus lateralis, Femoral, L2-L4					
8	+2	2.5 to 3 K	normal	normal	poor recruitment
Right, Tibialis anterior, Peroneal, L4-L5 s1					
7	+1	2.5 to 3 K	normal	normal	poor recruitment

The patient is undergoing continuous physical therapy and utilizes a knee-ankle-foot orthosis (KAFO) brace on both legs. As of the time of this report, he persists with an ambulatory delay, indicating a delay in the child's ability to walk independently. This is in contrast to motor delay, which encompasses delays in achieving gross motor milestones such as rolling over, sitting, crawling, standing, and walking.

## Discussion

Mutations in the TRPV4 gene can lead to abnormalities in bone, peripheral nerves, or both, potentially resulting in highly variable orthopedic and neurologic phenotypes [12]. The patient presents an uncommon homozygous mutation of the TRPV4 gene (c.281C>T; p.S94L) with a clinical presentation that differs from what has been previously reported in the literature [5]. Additionally, he exhibits congenital bilateral knee arthrogryposis associated with distal muscle weakness and bilateral talipes equinovarus, without vocal cord paralysis, torticollis, or skeletal abnormalities. Besides the symptoms previously described, he did not show any additional manifestations associated with diseases caused by TRPV4 mutations [4-7, 9-12].

In 2019, Velilla et al., on behalf of Brigham Genomics Medicine, reported a novel homozygous mutation in the TRPV4 gene (c.281C>T; p.S94L) in a patient presenting with vocal cord paralysis, torticollis, sensorineural hearing loss, diminished lower limb movement, spinal muscle atrophy, arthrogryposis multiplex congenita, severe skeletal abnormalities, and electrophysiologic findings suggestive of generalized motor axonopathy with coexisting denervation [5]. This unique homozygous variant TRPV4 mutation (c.281C>T; p.S94L) was termed the cause of the recessive form of congenital spinal muscular atrophy and arthrogryposis (CSMAA) [5].

Interestingly, our subject and Velilla's patient share the background of being born to unrelated healthy parents of Puerto Rican descent. Additionally, both patients experienced developmental ambulatory delay and were unable to stand or walk independently by the age of 1.5 years. Notably, both demonstrated normal fine and gross motor skills in their upper limbs. In contrast to Velilla's patient, our subject did not exhibit vocal cord paralysis, torticollis, sensorineural hearing loss, or skeletal dysplasia.

Genetically, both patients shared the same variant mutation in the TRPV4 gene (c.281C>T; p.S94L). This sequence alteration replaces serine with leucine at codon 94 of the TRPV4 protein [5]. Our patient represents the second case where this variant is present with a phenotypic manifestation of distal spinal muscle atrophy and arthrogryposis multiplex congenita. The homozygous c.281C>T (p.Ser94Leu) variant in the TRPV4 gene has now been identified in two independent occurrences in patients with CSMAA inherited from heterozygous unaffected carriers. This suggests the possibility of a founder effect. Whether a founder effect is likely to occur for this reportable disease in Puerto Rico would depend on several factors, including the prevalence of the disease in the original population, genetic diversity, and the size and genetic makeup of the founding population.

Previous studies have indicated that the pathogenic basis of the TRPV4 neuropathic spectrum is attributed to a gain of function, characterized by increased constitutive activity and heightened channel activation through mechanisms such as potentiated agonist activation [3, 8]. The mutated residues, contributing to this gain of function, hold structural significance and modulate gating functions [3, 10]. Their alteration results in heightened calcium influx both at resting and stimulated conditions, leading to cytotoxicity [3, 4, 6]. Functional studies have demonstrated that the TRPV4 (c.281C>T; p.S94L) variant leads to increased cytotoxicity, consistent with observations for other neuropathy-causing pathogenic TRPV4 variants [5].

TRPV4 mutations exhibit considerable variability in phenotypic expression, resulting in distinct clinical presentations [5]. It appears that genotype-phenotype correlations are not robust, and in the presence of a TRPV4 mutation, some allowance must be made for modulation of the clinical phenotype by other genes and/or non-genetic factors [5, 13, 14]. It is also believed that the region of the ankyrin repeat domain (ARD) where mutations are usually located will influence phenotypic variability, and different variations of the mutation can lead either to gain or loss of function [13].

Different mutations in the TRPV4 gene are associated with a spectrum of overlapping autosomal dominant conditions with either neuropathies or skeletal dysplasia; however, some patients exhibit both clinical phenotypes [5]. To date, more than 20 different mutations in TRPV4 have been identified in individuals affected by neuropathies and skeletal dysplasias [1]. An autosomal dominant mutation p.R315W was identified in a Dutch family with a phenotype of congenital distal spinal muscular atrophy (cDSMA) [6]. cDSMA is a clinically variable neuromuscular disorder characterized by a congenital lower motor neuron disorder restricted to the lower part of the body [6, 8]. Conversely, Auer-Grumbach et al. discovered two additional TRPV4 mutations (p.R269H and p.R316C) in affected members of three additional families with this phenotype [13].

Genetic analyses have revealed the presence of TRPV4 missense mutations at the R269H and R316C positions, associated with Charcot Marie Tooth disease Type 2 [6]. Charcot Marie Tooth disease Type 2 (CDCMT2C) is a disorder of the peripheral nervous system characterized by progressive muscle weakness and atrophy, initially affecting the peroneal muscles and later extending to the distal muscles of the arms, along with sensory abnormalities such as numbness, tingling, or loss of sensation [6, 12]. Neuropathies within the CDCMT2C group show signs of axonal regeneration in the absence of overt myelin alterations. Sensory and motor conduction assessments display a slight reduction in velocity and amplitude, while progressive muscle weakness and atrophy impact the distal regions of the body [6, 8].

A mutation in the p.R232C TRPV4 gene has been reported in a patient exhibiting scapuloperoneal spinal muscular atrophy (SPSMA), characterized by weakness of the scapularis muscle and bone abnormalities [2]. Hallmarks of SPSMA include shoulder girdle atrophy and weakness with scapular winging, combined with peroneal atrophy and findings of chronic denervation in the shoulder girdle and distal muscles [2].

Mutations at positions R616Q or V620I in the TRPV4 gene have been documented in the literature in patients exhibiting brachyolmia (BO) [6]. These mutations are situated in the fifth transmembrane region, a critical component of the functional pore [6]. Brachyolmia, an autosomal dominant condition, constitutes a clinically and genetically diverse group of skeletal dysplasias characterized by a short trunk, mild short stature, severe kyphoscoliosis, and flattened, irregular cervical vertebrae [6, 9].

Heterozygous mutations at positions K216E, G78W, K276E, and T740I in the TRPV4 gene have been reported by Dhanya et al. and Unger et al. as metatropic dysplasia (MTD), representing the most severe phenotypic spectrum within skeletal dysplasias [6, 7, 9, 15]. Metatropic Dysplasia is typified by short extremities, a truncated trunk with progressive kyphoscoliosis, rhizomelia, a coccygeal tail, and craniofacial abnormalities including a prominent forehead, midface hypoplasia, and a squared-off jaw [6, 16]. Additionally, TRPV4 p.I331F and p.P799L mutations are well-recognized inducers of MTD [6, 16].

## Conclusions

The literature review indicates that the correlation between phenotypic expression and TRPV4 mutation is not always uniform in its manifestations and may be contingent on the mutation's location, type, and presence of other genetic or environmental factors. This poses a challenge when attempting to explain and understand the mechanism by which similar molecular changes result in such diverse phenotypes. Due to this overlap in clinical and radiological features, it is essential to conduct mutation analysis for a definitive diagnosis. Identifying the specific gene variant is necessary for accurate disease prognosis, effective management, informed family planning, and the potential for personalized therapeutic interventions.
